# A Sensory Bias Has Triggered the Evolution of Egg-Spots in Cichlid Fishes

**DOI:** 10.1371/journal.pone.0025601

**Published:** 2011-10-18

**Authors:** Bernd Egger, Yuri Klaefiger, Anya Theis, Walter Salzburger

**Affiliations:** Zoological Institute, University of Basel, Basel, Switzerland; Biodiversity Insitute of Ontario - University of Guelph, Canada

## Abstract

Although, generally, the origin of sex-limited traits remains elusive, the sensory exploitation hypothesis provides an explanation for the evolution of male sexual signals. Anal fin egg-spots are such a male sexual signal and a key characteristic of the most species-rich group of cichlid fishes, the haplochromines. Males of about 1500 mouth-brooding species utilize these conspicuous egg-dummies during courtship – apparently to attract females and to maximize fertilization success. Here we test the hypothesis that the evolution of haplochromine egg-spots was triggered by a pre-existing bias for eggs or egg-like coloration. To this end, we performed mate-choice experiments in the basal haplochromine Pseudocrenilabrus multicolor, which manifests the plesiomorphic character-state of an egg-spot-less anal fin. Experiments using computer-animated photographs of males indeed revealed that females prefer images of males with virtual (‘in-silico’) egg-spots over images showing unaltered males. In addition, we tested for color preferences (outside a mating context) in a phylogenetically representative set of East African cichlids. We uncovered a strong preference for yellow, orange or reddish spots in all haplochromines tested and, importantly, also in most other species representing more basal lines. This pre-existing female sensory bias points towards high-quality (carotenoids-enriched) food suggesting that it is adaptive.

## Introduction

The haplochromines are the most famous and diverse group of cichlid fishes and widely distributed in Africa. Yet, their center of diversity is located in East Africa, where they constitute, for example, the entire cichlid species flocks of lakes Victoria and Malawi [1], [2], [3]. The actual species count for haplochromines remains unknown, although it is assumed that at least 1500 species are teeming in the lakes and rivers of East Africa [4], [5]. Save a small number of species, all haplochromines exhibit so-called egg-spots, making this trait *the* characteristic feature of haplochromines and a putative key innovation mediating their evolutionary success [1], [4]. The exceptions are several derived species that have lost egg-spots secondarily and a few basal species that presumably never had them [1].

**Figure 1:**
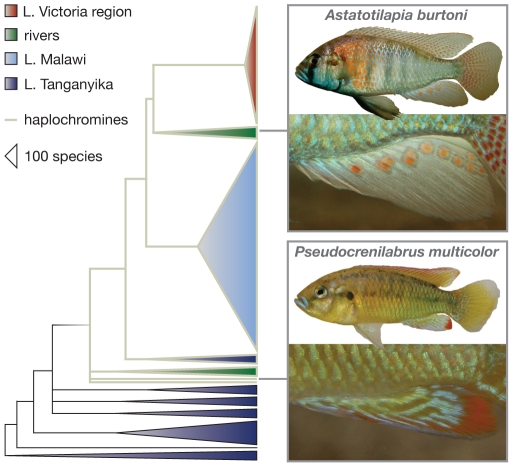
Schematic consensus phylogeny of the East African cichlids based on mitochondrial and nuclear gene segments (after [**1]**, [25], [36****]). The haplochromines (indicated by grey branches) are a derived and species-rich clade. The males of most haplochromine species display anal fin egg-spots, just as exemplified here for *Astatotilapia burtoni*. A few ancestral species, such as *Pseudocrenilabrus multicolor*, do not have egg-spots. Note that *A. burtoni* belongs to a riverine clade and occurs within Lake Tanganyika and surrounding rivers.

Genuine (‘true’) egg-spots are found on male anal fins and consist of a conspicuous yellow, orange, or reddish inner circle and a transparent outer ring (Figure 1) [3], [6], [7]. This makes them a costly trait, as fish cannot synthesize carotenoid-based pigments themselves [8], [9]. Egg-spots appear to resemble real eggs, which is why it has been proposed that these markings are ‘dummies’ that mimic freshly laid eggs in order to attract females and to maximize fertilization success [6], [7]. All haplochromines are female mouth-brooders, which means that females incubate their offspring – until fully developed – in their oral cavities. Immediately upon spawning, a haplochromine female takes up the eggs into her mouth; the territorial male instantly presents his anal fin egg-spots, to which the female responds in form of snatching, thereby positioning her mouth close to the males' genital papilla that discharges sperm. Wickler's egg mimicry hypothesis [6], [7] is disputed, however, as egg-spots often do not resemble size, shape and color of a species' actual eggs (see [10]). Also, it has been shown that fertilization success did not vanish when egg-spots were removed artificially [11], [12].

Here, we focus on the evolutionary origin of anal fin egg-spots rather than on their immediate function. More specifically, we test the hypothesis that the exploitation of a pre-existing bias has triggered the evolution of this conspicuous male trait in haplochromine cichlids [10]. The evolutionary origin of sexual signals is largely unknown and a matter of debate [14]. It is commonly accepted, however, that male signals can evolve in response to pre-existing sensory biases in females (‘sensory exploitation hypothesis’) [13], [14], [15], [16], [17], [18]. Such a female sensory bias may well be *adaptive*, namely if it evolved in another context than mating and through natural rather than through sexual selection [14], [17], [18]. Male guppies, for example, seem to mimic fruits that are a valuable food source and females are attracted by both males displaying the trait and by objects with respective colors [19]. Male swordtail characins, on the other hand, possess extended and pigmented opercular paddles that resemble invertebrate prey organisms [20]. Computer simulations also revealed that – at least under some circumstances – foraging preferences may result in increased mating preferences for similarly colored mates [21]. It has further been shown that disruptive female preferences in three-spine sticklebacks are linked to the visual systems' adaptation to different light regimes [22]. A similar case of ‘sensory drive speciation’ is reported from Lake Victoria haplochromines, where adaptations to different turbidity levels mediate female mate choice [23]. Finally, a preference for males with elaborated ornaments could also be adaptive in situations where males must ingest carotenoids to display these colors (e.g. [24]).

We find that females of a basal and egg-spot less haplochromine species prefer males with artificial (‘*in-silico*’) egg-spots and that haplochromines and more basal and non-mouth-brooding cichlid lines prefer color dots resembling egg-spots.

## Results

### Laboratory mate choice trials

**Figure 2:**
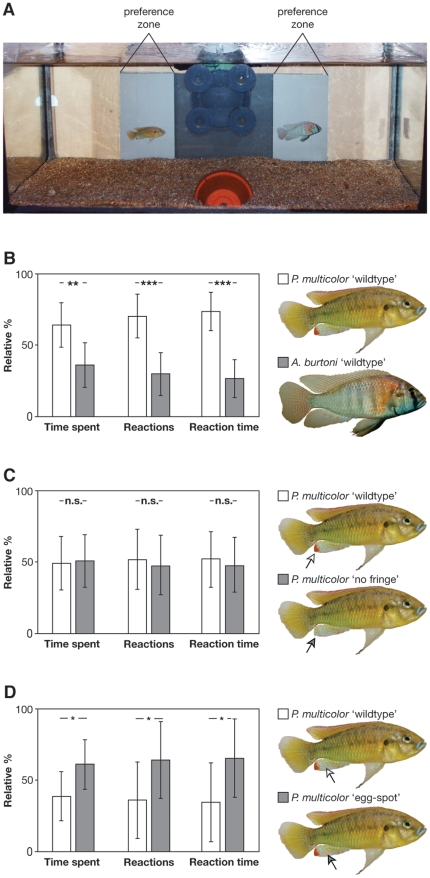
Female preference tests in *Pseudocrenilabrus multicolor* using computer animated stimuli. (A) The experimental set-up consists of an iMac computer behind an experimental aquarium (60×30×30 cm). Two animations are shown simultaneously (in this case a conspecific male and a heterospecific, *Astatotilapia burtoni*; see [B]). (B) Results from the ‘benchmark’ experiment, in which *P. multicolor* females were given the choice between a conspecific and a heterospecific (*A. burtoni*) male. The females reacted significantly more often with the animated image showing a conspecific male. (C) Results from the ‘red fringe’ experiments, in which *P. multicolor* were left the choice between a male with and one without the red fringe on the tip of the anal fin. We could not detect any difference in female response, which is also backed-up by two-way choice experiments with live fish (see Figure S1). (D) Results from the ‘egg-spot’ experiment, in which *P. multicolor* females could choose between a natural male and a male bearing an *in- silico* egg-spot. Females showed a significant preference for the male with the artificial egg-spot. Arrowheads indicate the minute differences between the images presented to the females.

We first tested whether females of the basal and egg-spot-less haplochromine cichlid *Pseudocrenilabrus multicolor* (Figure 1) could discriminate between males of their own species and males of another, more derived and egg-spot bearing haplochromine (*Astatotilapia burtoni*), when presented animated images on a computer screen in front of an experimental tank (Figure 2A). We found that focal females spent significantly more time and interacted significantly more often with the animation showing the conspecific male (related sample t-test; time spent: N = 12; t = 3.13; df = 11; p<0.01; number of reactions: N = 12; t = 4.72; df = 11; p<0.001; reaction time: N = 12; t = 6.06; df = 11; p<0.001) (see Figure 2B; Movie S1). Apart from demonstrating the females' ability to recognize conspecifics, this experiment highlights the usefulness of computer animations in female mate choice experiments with *P. multicolor*.

*Pseudocrenilabrus multicolor* females did not discriminate between animated images of males and such in which the red fin-fringe had been painted *in- silico* with the anal fin's brownish ground color (related sample t-test; time spent: N = 15; t = −0.17; df = 14; p = 0.87; number of reactions: N = 15; t = 0.38; df = 14; p = 0.71; reaction time: N = 15; t = 0.38; df = 14; p = 0.71; Figure 2C), suggesting that females are not advertent to the red fringe of male anal fins when choosing a mate. We confirmed this using live fish and a two-way choice set-up (time spent: related sample t-test; N = 15; t = 0.04; df = 14; p = 0.97; number of interactions: Wilcoxon signed-rank test; N = 15; V = 65; p = 0.78; interaction time: related sample paired t-test; N = 15; t = 0.05; df = 14; p = 0.96). This demonstrates that preference tests using computer animations reveal results congruent to mate choice experiments with live fish.

We found, however, that focal females spent significantly more time in front of the image of a male with the artificial egg-spot (Wilcoxon signed-rank test; N = 20; V = 41; p = 0.015); females also reacted more often with the egg-spot bearing male by following its animated movements (related sample t-test; N = 20; t = −2.35; df = 19; p = 0.029); and, *P. multicolor* females spent more time reacting with the image of a modified male (Wilcoxon signed-rank test; N = 20; V = 42.5; p = 0.020) (Figure 2D). This clearly indicates that females of an ancestral haplochromine species show a preference for males with the derived character state of egg-spots, which is suggestive for the existence of a pre-existing bias for orange spots.

### Color-dot preference tests

**Figure 3:**
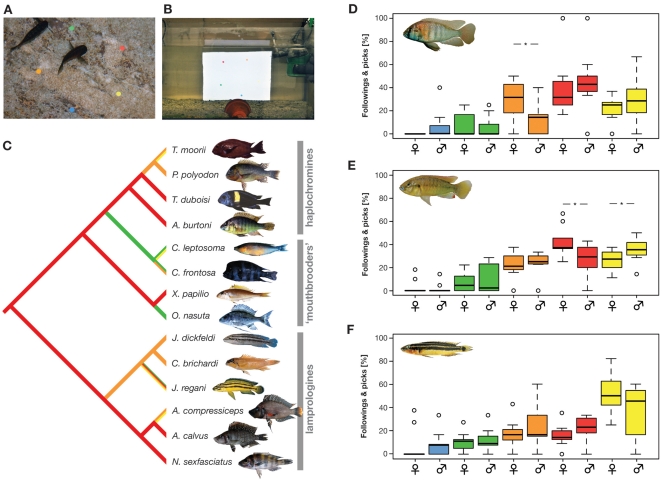
Color preference tests in different East African cichlid species. (A) Set-up of the field experiment at Lake Tanganyika. Fishes were presented five color dots on a transparent foil and we measured the number of pecks towards each dot. (B) Set-up of the laboratory experiments. Individual fishes were presented five color dots on a computer screen. (C) Ancestral character state reconstruction of color preferences in a phylogenetically representative set of cichlids from Lake Tanganyika. Most species clearly preferred orange or red colors. Importantly, also the substrate spawning lamprologines showed such a preference. (D–F) Results from the color-dot preference experiments in the laboratory with the haplochromines Astatotilapia burtoni (D) and Pseudocrenilabrus multicolor (E) and the lamprologine Julidochromis ornatus (F). Significant differences between males and females are indicated.

In our color-dot experiments in the field, all four tested haplochromine species showed a strong preference for yellow, orange or red dots (Tables S1, S2). Importantly, most other species belonging to basal cichlid lineages did so, too, and only three species showed a weak (*C. frontosa* and *C. leptosoma*) or strong (*O. nasuta*) preference for green. Notably, *C. frontosa* reacted almost as often to orange dots (29 times) as it did to green ones (30 times); a similar situation was observed for *C. leptosoma* between yellow (8 times) and green (11 times). For both species, a clear preference could thus not be determined. Also, with only 20 pecks each in a period of five minutes, *C. leptosoma* and *O. nasuta* showed the by far smallest number of pecks, questioning the strength of their preference for a particular color. In any case, a character state reconstruction on the basis of a molecular phylogeny (Figure 3C) clearly indicates that the preference for red dots existed before the evolution of haplochromines, irrespective of how we coded the preference for *C. frontosa*, *C. leptosoma* and *O. nasuta* (indecisive, orange or green, yellow or green).

In the laboratory experiments using computer animated color dots (Figure 3B, D–F), we detected a non-random distribution of color preferences in all three species tested (Friedman test; *A. burtoni*, N = 20; p<0.001; *P. multicolor*, N = 20; p<0.001; *J. marlieri*, N = 20; p<0.001).

In line with our color preference experiments in the field, all three species showed a preference for egg-spot like colors (yellow, orange and red), while blue and green were hardly ever chosen (Figure 3D–F, Table S3). Importantly, *A. burtoni*, which is the only species that we could test both in the field and in the lab, showed highly congruent responses to the stationary color dots in the pond set-up and the animated color dots in the laboratory experiments. Interestingly our lab experiments uncovered sex-specific differences in the haplochromines: *A. burtoni* females significantly more often pecked at and followed the orange-colored dots (Wilcoxon rank-sum test; N = 20; p = 0.037) and *P. multicolor* females significantly more often pecked at and followed the red-colored dots than did the males (Wilcoxon rank-sum test; N = 20; p = 0.045), while *P. multicolor* males reacted more often to yellow dots compared to females (Wilcoxon rank-sum test; N = 20; p = 0.045).

## Discussion

Anal fin egg-spots are a characteristic feature of the most species-rich group of cichlids, the haplochromines [1], [4], [25]. While several hypothesis exist that seek to explain the function of this conspicuous male trait (see e.g. [6], [7], [12]), little is known about their evolutionary origin. Here we test the hypothesis that male egg-spots in haplochromines evolved to exploit a pre-existing bias in females [10]. A crucial prerequisite in favor of this hypothesis is that the preference for egg-spots (the sensory bias) is phylogenetically older than anal fin egg-spots themselves [14], [18], [26], [27]. We confirm this prediction in two independent and *per se* complementary experiments.

First, we show that females of the basal haplochromine species *Pseudocrenilabrus multicolor*, which manifests the plesiomorphic character-state of an egg-spot-less anal fin (Figure 1), do show a clear preference for the animated photograph of a male with an artificial egg-spot over an otherwise identical animated photograph of a male without an egg-spot (Figure 2D). Obviously, *P. multicolor* females perceive the minute difference between the two computer-animated images of males (i.e. the artificial egg-spot, which spans less than 1% of the lateral area), which seems plausible given the visual capabilities of cichlids [17], [18].

Second, our field experiments suggest that a preference for yellow, orange or red dots, which resemble the color and shape of egg-spots, existed before the radiation of the haplochromines. Most East African cichlid species tested and, importantly, the majority of the egg-spot-less species belonging to cichlid lineages basal to the haplochromines, show clear preferences for such egg-spot-like dots over blue and green dots (Table S1, Figure 3). The only three species not showing a clear preference for egg-spot-like colors were indecisive and/or showed very weak preferences overall (as measured by the number of pecks per 5 minute trial). Our character state reconstructions indicate that the preference for egg-spot-like colors was present before the emergence of the first haplochromines and that even the substrate spawning lamprologines show a bias towards yellow, orange or red dots (Figure 3C). These results are backed up by our color preference experiments under laboratory conditions in two haplochromines and one lamprologine (Figure 3D–F).

Taken together, our experiments suggest that sensory exploitation of a pre-existing bias was responsible for the evolution of anal fin egg-spots in haplochromine cichlids. The question is now what could have triggered the bias for egg-spot-like dots in (female) cichlids. Tobler [10] proposed that it is the affinity to detect own eggs as such. This should have evolved in mouth-brooding females as a result of their limited number of relatively large eggs and, consequently, the immediate reduction of fitness when failing to take up all the eggs. This hypothesis is compatible with our mate choice experiments in the basal and egg-spot-less haplochromine *P. multicolor*. Yet, the preference for egg-spot-like dots is prevalent in male and female cichlids and also in substrate spawners basal to haplochromines (which, nevertheless, perform brood care). This, in turn, suggests that it is not the affinity for own eggs that evolved, as males should not show this affinity and substrate spawners have much smaller and less conspicuous eggs. It seems more likely that the observed pre-existing bias in East African cichlids points towards high quality – e.g. carotenoid-rich – food like shrimps, algae and, notably, fish eggs. A preference for carotenoid-enriched diets is known from several taxa (e.g. [28], [29], [30]), and the heritability of algal-foraging ability in guppies suggests that, in this case, females might actually benefit from preferring males with a pronounced carotenoid-based coloration indicative of their foraging skills [29], [31]. Such a pre-existing bias towards yellow, orange or reddish dots that resemble food could reasonably well explain why yellow, orange or reddish egg-spots (i.e. convergently evolved blotches on the fins of other cichlids [4], [10], [25]) have evolved multiple times in addition to and outside the haplochromines.

## Methods

### Laboratory mate choice trials

All laboratory mate choice experiments were performed at the Zoological Institute of the University of Basel under the permission of the Cantonal Veterinary Office, Basel, Switzerland (permit number 2403). Live cichlids were kept in isolation and under standardized conditions (12 h dark/12 h light; 25°C).

Before turning towards our central question, we had to assess the usefulness of computer animations in experiments with the haplochromine cichlid species *Pseudocrenilabrus multicolor*. While computer-animated stimuli are frequently used in West African cichlids [32], [33], little is known about how haplochromines react to it. Finally, there is a technical component too, as it has been shown that the reaction to a stimulus may vary depending on the computer screen used [34]. Therefore, we first tested three different computer screens: a SONY® 17″ CRT display, and two Apple® iMac computers with a dull 17″ and a bright 21″ LCD display, respectively. In our set-up, females reacted most when presented images on the 17″ iMac G5 (pers. observation). We also evaluated still and animated photographs of males and found that female *P. multicolor* reacted most to the following animations: 7 seconds upwards movement, 2 seconds remaining in still position, 7 seconds downward movement, 2 seconds remaining in still position (pers. observation; the animations were created with Apple® Keynote® software and exported as Quicktime® movies).

As a benchmark, we tested whether *P. multicolor* females can discriminate between a conspecific and a heterospecific (*Astatotilapia burtoni*) male. To this end, we positioned an iMac (17″ iMac G5 running Mac OSX version 10.5.7; chip model ATY Radeon×1600, 1400×900 pixels, 32 Bit color) directly behind a glass aquarium (60×30×30 cm) so that it covered about 2/3 of the aquarium's width (Figure 2A). On the very left and the very right of the iMac, there was a 10.5 cm neutral zone not covered by the screen. These areas plus the two sides were covered with visual barriers, so that only the front panel remained transparent. Thus, we could video-tape each experimental trial with a SONY® DCR-HC90E Handycam® (note that all computer- animated experiments were performed in a closed compartment to avoid interference of the experimenter). The bottom of the aquarium was covered with sand, and in the front center, right below the filter, we placed half a flower-pot to provide shelter to the focal female. For the animations, the screen was divided into two 10.5 cm wide outer parts (where the actual animations were shown) and an 18 cm central part that remained grey (Figure 2A). In this experiment, twelve *P. multicolor* females were exposed to two size-matched images of a male *P. multicolor* and a male *A. burtoni*, which were animated to move up and down in an infinite loop (see above for animation settings); the images of the males were pasted into a neutral grey background (R: 149, G: 149, B: 149). Each female was tested twice, once in the morning and once in the afternoon (with at least 5 h between experiments), and the stimuli were switched between the two rounds (with the morning set-up being chosen randomly). At the beginning of each experiment, the female was allowed to habituate for 10 minutes before the parallel animations started. Beginning from the first reaction of the focal female to the animation (i.e. the female swimming towards the animation, stopping in front of the monitor, facing the stimulus and swimming along with the animation), we recorded the following three – not mutually exclusive – behavioral parameters for a period of ten minutes (based on the video-taped material): (*i*) ‘*time spent*’ (in seconds) as the time that a female spent in front of each animation (practically, we started counting when 50% of the female body entered the preference zone, i.e. the 10.5 cm grey zone of each animated male, and stopped when 50% of the female body left this zone); (*ii*) ‘*number of reactions*’ (integer) in how often a given female would follow the up- or downward-movement of a stimulus male; and (*iii*) ‘*reaction time*’ as the time (in seconds) that a female would actively follow the up- or downward-movement of a stimulus male. For statistical analyses, the counts from the two rounds of experiments with each focal female were averaged. To account for individual differences in the total time spent and the number of reactions among females, we used individual percentages of the total number of observations as response variables [32], [34]. All data were analyzed with the software R (vers. 2.8.1).

In a second round of experiments, we focused on the red fringe on the anal fin of male *P. multicolor*, as we could not exclude the possibility that this trait is the target of female choice in this basal haplochromine species. We used the same parameters as before, except that this time we gave females the choice between two images of a male, of which one retained the natural phenotype, whereas the other was modified *in-silico* so that its red fringe was replaced by the brownish ground color of the rest of the anal fin (using Adobe® Photoshop®). We tested fifteen focal females and recorded the very same behavioral parameters as mentioned above.

We then repeated this experiment with live fish using a dichotomous set-up (Figure S1A): six pairs of size-matched males of *P. multicolor* were formed to avoid bias. The red fringe on the anal fin of one male of each size-matched pair was removed by fin-clipping. On the other male a piece of dorsal fin was cut to control for possible treatment effects (Figure S1B). The size-matched males of each pair were randomly placed in one of the two outer tanks (40×24×24 cm) adjacent to a central tank (60×30×30 cm). The males were allowed to habituate for several days; during this period the males were inspected for signs of stress. Then, a focal female was placed into the central tank. We recorded the following parameters during 10-minute trials starting with the first interaction: (*i*) ‘*time spent*’ (in seconds) as the time that a female spent in a preference zone (12 cm adjacent to each male tank); (*ii*) ‘*interactions’* as the number of independent visits to a preference zone; and (*iii*) ‘*interaction time*’ as the time (in seconds) that a female spent in front of an interacting male.

Finally, we tested for a pre-existing bias for egg-spots in females of *P. multicolor* using computer animated stimuli. We presented females two identical male images, except that one of them had an artificial egg-spot. This single egg-spot was designed to resemble real *P. multicolor* eggs in color and average size. Therefore, we photographed and measured 46 eggs and determined the average size (1.86 mm) and color hue (R: 255, G: 150, B: 45). This ‘average’ egg-spot was then pasted onto the anal fin of a male image using Photoshop®.

### Color-dot preference tests

#### Pond experiments

The preference tests for egg-spot-like dots were carried out in February and March 2010 at ‘Kalambo Lodge’ at the shore of Lake Tanganyika, East Africa (Zambia; S 8.6232 E 31.2). Wild-caught individuals from 14 cichlid species were kept in ponds (ca. 1×2 m) filled with lake water (ca. 50 cm high). We tested four egg-spot bearing haplochromine species (*Astatotilapia burtoni*, *Petrochromis polyodon*, *Tropheus duboisi* and *T. moorii*) and ten species belonging to other, more basal cichlid lineages including mouth-brooding (*Cyphotilapia frontosa*, *Cyprichromis leptosoma*, *Ophthalmotilapia nasuta* and *Xenotilapia papilio*) and substrate spawning (*Altolamprologus calvus*, *A. compressiceps*, *Chalinochromis brichardi*, *Julidochromis dickfeldi*, *J. regani* and *Neolamprologus sexfasciatus*) representatives. Each pond contained between 11 and 75 individuals, depending on the size of the fish and the sampling success of the local fishermen. All ponds were stocked with a mix of female and male individuals. As most species under study do not show sexual dimorphisms, the exact sex ratio could not be determined. To the 14 species, we presented five conspicuous color dots (yellow, orange, red, green, and blue), which were arranged in a pentagonal shape on a transparent foil (Figure 3A). Two sets of foils with different arrangements of dots were used. After placing the foil on the ponds' grounds, we waited until the first individual approached and pecked at one of the dots. Four observers then counted the number of pecks for a period of five minutes. If one individual stayed at one spot and pecked at it repeatedly, it was counted as one strike only. We first performed a goodness-of-fit test to examine the existence of a preference for certain colors within species (all species preferred some colors over others; p<0.001; Table S1). The color preference within each species was then determined using a series of binomial tests (Table S2) and subjected to an ancestral character state reconstruction. To this end, we used a phylogenetic tree derived from a maximum likelihood analysis based on mitochondrial sequence data (NADH Dehydrogenase Subunit II gene; 1047 bp; [1], [35]). Preference for the colors blue, green, yellow, orange and red were coded as numbers and we allowed for multiple characters states in species that did not show a significant preference for only one color. Ancestral color preferences were reconstructed with parsimony as implemented in Mesquite (vers. 2.74, [36]). We would like to note here that it is essentially impossible to perform such an experiment within the lake itself, as there are too many species and interactions between species; also, we would never find so many individuals of the same species together. It is also important to note that we were not able to test *P. multicolor* in the wild, as this species does not occur within Lake Tanganyika.

#### Laboratory experiments

Since the color-dot preference tests in the field could potentially be influenced by pseudo-replication within ponds, we repeated this experiment in the lab using three available lab strains and computer animations. Three species (*Pseudocrenilabrus multicolor*, 10 males and 10 females; *Astatotilapia burtoni*, 11 males and 9 females; *Julidochromis ornatus*, 9 males and 11 females) were tested for color preference under controlled laboratory conditions, allowing assessing individual fish and males and females separately. To this end, five colored spots (yellow, orange, red, green, and blue; diameter: 1 cm) were arranged circularly on neutral grey background in a computer animation, displaying a simultaneous circular movement. Two animations were designed to randomize the initial position of the five color dots. The focal fish was introduced into an aquaria tank (60×30×30 cm) and left for 30 min before the start to acclimatize. Then the animation was presented to the focal fish via a computer screen (see above), placed in front of the experimental tank. The behavior of the focal fish was recorded for 1 hour with a video-camera and analyzed with the software iMovie®. Thirty minutes of behavior after the first reaction were analyzed and two parameters were recorded: the number of times the focal fish pecked each colored dot and number of times the focal fishes followed each colored dot. The percentage data was angular-transformed and analyzed with the statistics software R, applying a Friedman test and a series of Wilcoxon signed-rank tests (with and without Bonferroni correction; Table S3). Sex differences were tested through Wilcoxon rank-sum tests.

## Supporting Information

Figure S1 **Two-way choice tests in** ***Pseudocrenilabrus multicolor*** **.** (A) Scheme of the experimental set-up consisting of two outer tanks (40×24×24 cm) adjacent to a central tank (60×30×30 cm). Each male tank (outer tanks) was equipped with a plastic perforated shelter, while the central female tank was equipped with three shelters: two shelters were placed next to each outer male tank and one shelter was placed in the middle of the tank. In this setup the females had the possibility to communicate visually with the two different males at the left and right extreme of their central tank (12 cm preference zone). Only visual communication was permitted. (B) Results from the ‘fin-clipping’ experiment, in which *P. multicolor* females were given the choice between a male where the red fringe at the anal fin was removed by fin-clipping and a size-matched control male that was fin-clipped at the dorsal fin. Females did not show any preference.
(PDF)

Table S1 Color-dot preference tests in ponds. Preferred colors for each species are indicated.
(PDF)

Table S2 Color-dot experiments in ponds. P-values resulting from binomial tests.
(PDF)

Table S3 Laboratory color-dot preference test. P-values were calculated from percentage data with arcsine transformation and are presented with and without Bonferroni correction for *Astatotilapia burtoni* (A), *Pseudocrenilabrus multicolor* (B) and *Julidochromis ornatus* (C).
(PDF)

Movie S1 Female choice experiments in *Pseudocrenilabrus multicolor* using computer animations.
(MOV)

## References

[1] Salzburger W, Mack T, Verheyen E, Meyer A (2005). Out of Tanganyika: Genesis, explosive speciation, key-innovations and phylogeography of the haplochromine cichlid fishes.. BMC Evol Biol.

[2] Verheyen E, Salzburger W, Snoeks J, Meyer A (2003). Origin of the superflock of cichlid fishes from Lake Victoria, East Africa.. Science.

[3] Fryer G, Iles TD (1972). The cichlid fishes of the Great Lakes of Africa: Their biology and Evolution.

[4] Salzburger W (2009). The interaction of sexually and naturally selected traits in the adaptive radiations of cichlid fishes.. Mol Ecol.

[5] Kocher TD (2004). Adaptive evolution and explosive speciation: the cichlid fish model.. Nature Rev Genet.

[6] Wickler W (1962). ‘Egg-dummies’ as natural releasers in mouth-breeding cichlids.. Nature.

[7] Wickler W (1962). Zur Stammesgeschichte funktionell korrelierter Organ- und Verhaltensmerkmale: Ei-Attrappen und Maulbrüten bei afrikanischen Cichliden.. Z Tierpsychol.

[8] Lozano GA (1994). Carotenoids, parasites, and sexual selection.. Oikos.

[9] Grether GF, Hudon J, Endler JA (2001). Carotenoid scarcity, synthetic pteridine pigments and the evolution of sexual coloration in guppies (Poecilia reticulata).. Proc R Soc Lond B Biol Sci.

[10] Tobler M (2006). The eggspots of cichlids: Evolution through sensory exploitation?. Zeitschrift für Fischkunde.

[11] Hert E (1991). Female choice based on egg-spots in *Pseudotropheus aurora* Burgess 1976, a rock-dwelling cichlid of Lake Malawi, Africa.. J Fish Biol.

[12] Hert E (1989). The function of egg-spots in an African mouth-brooding cichlid fish.. Anim Behav.

[13] Endler JA, McLellan T (1988). The process of evolution: towards a newer synthesis.. Annu Rev Ecol Syst.

[14] Arnqvist G (2006). Sensory exploitation and sexual conflict.. Philos Trans R Soc Lond B Biol Sci.

[15] West-Eberhard MJ (1979). Sexual selection, social competition, and evolution.. P Am Philos Soc.

[16] Ryan MJ (1990). Sexual selection, sensory systems, and sensory exploitation.. Oxf Surv Evol Biol.

[17] Boughman JW (2002). How sensory drive can promote speciation.. Trends Ecol Evol.

[18] Kirkpatrick M, Ryan MJ (1991). The evolution of mating preferences and the paradox of the lek.. Nature.

[19] Rodd FH, Hughes KA, Grether GF, Baril CT (2002). A possible non-sexual origin of mate preference: are male guppies mimicking fruit?. Proc R Soc Lond B Biol Sci.

[20] Arnqvist G, Kolm N (2010). Population differentiation in the swordtail characin (Corynopoma riisei): a role for sensory drive?. J Evol Biol.

[21] Fuller RC (2009). A test of the critical assumption of the sensory bias model for the evolution of female mating preference using neural networks.. Evolution.

[22] Boughman JW (2001). Divergent sexual selection enhances reproductive isolation in sticklebacks.. Nature.

[23] Seehausen O, Terai Y, Magalhaes IS, Carleton KL, Mrosso HD (2008). Speciation through sensory drive in cichlid fish.. Nature.

[24] Grether GF, Kolluru GR, Rodd FH, de la Cerda J, Shimazaki K (2005). Carotenoid availability affects the development of a colour-based mate preference and the sensory bias to which it is genetically linked.. Proc R Soc Lond B Biol Sci.

[25] Salzburger W, Braasch I, Meyer A (2007). Adaptive sequence evolution in a color gene involved in the formation of the characteristic egg-dummies of male haplochromine cichlid fishes.. BMC Biol.

[26] Endler JA, Basolo AL (1998). Sensory ecology, receiver biases and sexual selection.. Trends Ecol Evol.

[27] Ryan MJ (1998). Sexual selection, receiver biases, and the evolution of sex differences.. Science.

[28] Senar JC, Moller AP, Ruiz I, Negro JJ, Broggi J (2010). Specific appetite for carotenoids in a colorful bird.. PLoS ONE.

[29] Karino K, Utagawa T, Shinjo S (2005). Heritability of the algal-foraging ability: an indirect benefit of female mate preference for males' carotenoid based coloration in the guppy, *Poecilia reticulata*.. Behav Ecol Sociobiol.

[30] Milinski M, Bakker TC (1990). Female sticklebacks use male coloration in mate choice and hence avoid parasitized males.. Nature.

[31] Karino K, Shinjo S (2007). Relationship between algal-foraging ability and expression of sexually selected traits in male guppies.. Zool Sci.

[32] Baldauf SA, Kullmann H, Schroth SH, Thunken T, Bakker TC (2009). You can't always get what you want: size assortative mating by mutual mate choice as a resolution of sexual conflict.. BMC Evol Biol.

[33] Baldauf SA, Bakker TCM, Kullmann H, Thunken T (2011). Female nuptial coloration and its adaptive significance in a mutual mate choice system.. Behav Ecol.

[34] Baldauf SA, Kullmann H, Thunken T, Winter S, Bakker TC (2009). Computer animation as a tool to study preferences in the cichlid Pelvicachromis taeniatus.. J Fish Biol.

[35] Salzburger W, Meyer A, Baric S, Verheyen E, Sturmbauer C (2002). Phylogeny of the Lake Tanganyika cichlid species flock and its relationship to the Central and East African haplochromine cichlid fish faunas.. Syst Biol.

[36] Maddison WP, Maddison DR (2005). Mesquite: A modular system for evolutionary analysis. 1.06 ed.. http://mesquiteproject.org.

