# Supplementary figures and images for: A Sensory Bias Has Triggered the Evolution of Egg-Spots in Cichlid Fishes
Source: PLoS One. 2011 Oct 18;6(10):e25601. doi: 10.1371/journal.pone.0025601 (PMC3196499; doi:10.1371/journal.pone.0025601)

**A**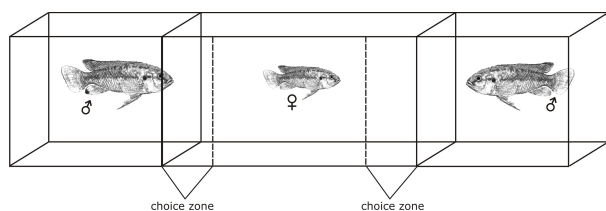**B**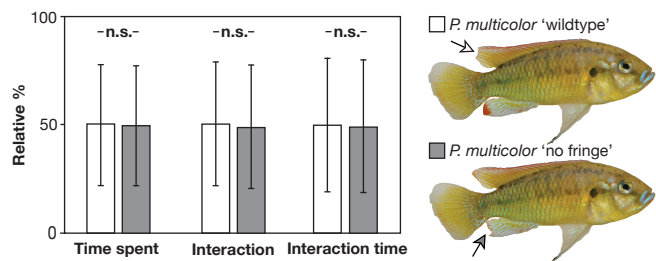

Supplement: Figure S1 — Two-way choice tests in Pseudocrenilabrus multicolor . (A) Scheme of the experimental set-up consisting of two outer tanks (40×24×24 cm) adjacent to a central tank (60×30×30 cm). Each male tank (outer tanks) was equipped with a plastic perforated shelter, while the central female tank was equipped with three shelters: two shelters were placed next to each outer male tank and one shelter was placed in the middle of the tank. In this setup the females had the possibility to communicate visually with the two different males at the left and right extreme of their central tank (12 cm preference zone). Only visual communication was permitted. (B) Results from the ‘fin-clipping’ experiment, in which P. multicolor females were given the choice between a male where the red fringe at the anal fin was removed by fin-clipping and a size-matched control male that was fin-clipped at the dorsal fin. Females did not show any preference. (PDF) [file pone.0025601.s001.pdf]
