# Supplementary material for: A Sensory Bias Has Triggered the Evolution of Egg-Spots in Cichlid Fishes
Source: PLoS One. 2011 Oct 18;6(10):e25601. doi: 10.1371/journal.pone.0025601 (PMC3196499; doi:10.1371/journal.pone.0025601)
Supplement: Table S1 — Color-dot preference tests in ponds. Preferred colors for each species are indicated. (PDF) [file pone.0025601.s002.pdf]

species	sample size	yellow	orange	red	blue	green	goodness of fit
Altolamprologus calvus	45	6	21	162	0	1	p<0.001
Altolamprologus compressiceps	50	77	104	11	2	10	p<0.001
Astatotilapia burtoni	36	11	43	154	0	0	p<0.001
Chalinochromis brichardi	45	13	31	10	7	2	p<0.001
Cyphotilapia frontosa	43	14	29	11	6	30	p<0.001
Cyprichromis sp. leptosoma	50	8	0	1	0	11	p<0.001
Julidochromis dickfeldi	30	3	75	33	0	9	p<0.001
Julidochromis regani	27	11	13	20	0	9	p<0.001
Neolamprologus sexfasciatus	18	3	2	62	0	24	p<0.001
Ophtalmotilapia nasuta	12	2	4	0	0	14	p<0.001
Petrochromis polyodon	11	17	48	16	1	2	p<0.001
Tropheus duboisi	70	67	169	250	6	18	p<0.001
Tropheus moorii	30	22	32	4	0	2	p<0.001
Xenotilapia papilio	75	1	8	31	0	1	p<0.001
