# Supplementary material for: A Sensory Bias Has Triggered the Evolution of Egg-Spots in Cichlid Fishes
Source: PLoS One. 2011 Oct 18;6(10):e25601. doi: 10.1371/journal.pone.0025601 (PMC3196499; doi:10.1371/journal.pone.0025601)
Supplement: Table S2 — Color-dot experiments in ponds. P-values resulting from binomial tests. (PDF) [file pone.0025601.s003.pdf]

**binomial test**	*A. calvus*	*A. compressiceps*	*A. burtoni*	*C. brichardi*	*C. frontosa*	*C. sp. leptosoma*	*J. dickfeldi*	*J. regani*	*N. sexfasciatus*	*O. nasuta*
yellow vs.orange	0.0059	0.0530	< 0.0001	0.0096	0.0315	0.0078	< 0.0001	0.8388	1	0.6875
yellow vs. red	< 0.0001	< 0.0001	< 0.0001	0.6776	0.6900	0.0391	< 0.0001	0.1496	< 0.0001	0.5000
yellow vs. blue	0.0313	< 0.0001	0.0010	0.2632	0.1153	0.0078	0.2500	0.0010	0.2500	0.5000
yellow vs. green	0.1250	< 0.0001	0.0010	0.0074	0.0226	0.6476	0.1460	0.8238	< 0.0001	0.0042
orange vs. red	< 0.0001	< 0.0001	< 0.0001	0.0015	0.0064	-	< 0.0001	0.2962	< 0.0001	0.1250
orange vs. blue	< 0.0001	< 0.0001	< 0.0001	0.0001	0.0001	-	< 0.0001	0.0002	0.5000	0.1250
orange vs. green	< 0.0001	< 0.0001	< 0.0001	< 0.0001	1	0.0010	< 0.0001	0.5235	< 0.0001	0.0309
red vs. blue	< 0.0001	0.0225	< 0.0001	0.6291	0.3323	-	< 0.0001	< 0.0001	< 0.0001	-
red vs. green	< 0.0001	1	< 0.0001	0.0386	0.0043	0.0063	0.0003	0.0614	< 0.0001	0.0001
blue vs. green	-	0.0386	-	0.1797	< 0.0001	0.0010	0.0039	0.0039	< 0.0001	0.0001

P. polyodon	*T. duboisi*	*T. moorii*	*X. papilio*
0.0065	< 0.0001	0.2203	0.0391
1	< 0.0001	0.0005	< 0.0001
0.0001	< 0.0001	< 0.0001	-
0.0007	< 0.0001	< 0.0001	1.5
< 0.0001	< 0.0001	< 0.0001	0.0003
< 0.0001	< 0.0001	< 0.0001	0.0078
< 0.0001	< 0.0001	< 0.0001	0.0391
0.0003	< 0.0001	0.1250	< 0.0001
0.0013	< 0.0001	0.6875	< 0.0001
1	0.0227	0.5000	-
