# Supplementary material for: A Sensory Bias Has Triggered the Evolution of Egg-Spots in Cichlid Fishes
Source: PLoS One. 2011 Oct 18;6(10):e25601. doi: 10.1371/journal.pone.0025601 (PMC3196499; doi:10.1371/journal.pone.0025601)
Supplement: Table S3 — Laboratory color-dot preference test. P-values were calculated from percentage data with arcsine transformation and are presented with and without Bonferroni correction for Astatotilapia burtoni (A), Pseudocrenilabrus multicolor (B) and Julidochromis ornatus (C). (PDF) [file pone.0025601.s004.pdf]

A1 *Astatotilapia burtoni* - males and females (n = 20)

Friedman test

parameter	p-value
pecks & chases	4.38 *10-8

Wilcoxon signed-rank test

parameter	p-value	significant? (without Bonferroni correction)		significant? (with Bonferroni correction)	
**pecks**					
red-blue	0.0005199	< 0.05	yes	< 0.05/10	yes
yellow-blue	0.0006637	< 0.05	yes	< 0.05/9	yes
red-green	0.0006807	< 0.05	yes	< 0.05/8	yes
yellow-green	0.000832	< 0.05	yes	< 0.05/7	yes
orange-green	0.00909	< 0.05	yes	> 0.05/6	no
orange-blue	0.01379	< 0.05	yes	> 0.05/5	no
orange-red	0.04538	< 0.05	yes	> 0.05/4	no
orange-yellow	0.09316	> 0.05	no	> 0.05/3	no
red-yellow	0.6085	> 0.05	no	> 0.05/2	no
blue-green	1	> 0.05	no	> 0.05/1	no
**chases**					
red-green	0.001075	< 0.05	yes	< 0.05/10	yes
red-blue	0.004363	< 0.05	yes	< 0.05/9	yes
red-yellow	0.02075	< 0.05	yes	> 0.05/8	no
orange-red	0.03242	< 0.05	yes	> 0.05/7	no
orange-blue	0.07065	> 0.05	no	> 0.05/6	no
yellow-blue	0.1091	> 0.05	no	> 0.05/5	no
orange-green	0.1815	> 0.05	no	> 0.05/4	no
orange-yellow	0.3271	> 0.05	no	> 0.05/3	no
blue-green	0.3523	> 0.05	no	> 0.05/2	no
yellow-green	0.673	> 0.05	no	> 0.05/1	no
**pecks & chases**					
yellow-blue	0.0003143	< 0.05	yes	< 0.05/10	yes
red-green	0.0003173	< 0.05	yes	< 0.05/9	yes
red-blue	0.0004447	< 0.05	yes	< 0.05/8	yes
yellow-green	0.002072	< 0.05	yes	< 0.05/7	yes
orange-blue	0.008514	< 0.05	yes	> 0.05/6	no
orange-red	0.009677	< 0.05	yes	> 0.05/5	no
orange-green	0.02185	< 0.05	yes	> 0.05/4	no
red-yellow	0.1146	> 0.05	no	> 0.05/3	no
orange-yellow	0.5319	> 0.05	no	> 0.05/2	no
blue-green	0.2195	> 0.05	no	> 0.05/1	no

A2 *Astatotilapia burtoni* - females only (n = 9)

Friedman test

parameter	p-value
pecks	0.002
chases	3.90*10-4
pecks & chases	2.61*10-4

Wilcoxon signed-rank test

parameter	p-value	significant? (without Bonferroni correction)		significant? (with Bonferroni correction)	
**pecks**					
yellow-blue	0.02154	< 0.05	yes	> 0.05/10	no
yellow-green	0.02154	< 0.05	yes	> 0.05/9	no
red-blue	0.03552	< 0.05	yes	> 0.05/8	no
red-green	0.03552	< 0.05	yes	> 0.05/7	no
orange-blue	0.05791	> 0.05	no	> 0.05/6	no
orange-green	0.05791	> 0.05	no	> 0.05/5	no
orange-yellow	0.4974	> 0.05	no	> 0.05/4	no
red-yellow	0.6845	> 0.05	no	> 0.05/3	no
orange-red	1	> 0.05	no	> 0.05/2	no
blue-green	NA	> 0.05	no	> 0.05/1	no
**chases**					
red-blue	0.008849	< 0.05	yes	> 0.05/10	no
orange-blue	0.02154	< 0.05	yes	> 0.05/9	no
red-yellow	0.02225	< 0.05	yes	> 0.05/8	no
red-green	0.03552	< 0.05	yes	> 0.05/7	no
orange-yellow	0.05149	> 0.05	no	> 0.05/6	no
orange-green	0.2016	> 0.05	no	> 0.05/5	no
yellow-blue	0.1003	> 0.05	no	> 0.05/4	no
blue-green	0.1003	> 0.05	no	> 0.05/3	no
orange-red	0.4461	> 0.05	no	> 0.05/2	no
yellow-green	0.7525	> 0.05	no	> 0.05/1	no
**pecks & chases**					
red-blue	0.00909	< 0.05	yes	> 0.05/10	no
yellow-blue	0.01379	< 0.05	yes	> 0.05/9	no
orange-blue	0.02201	< 0.05	yes	> 0.05/8	no
red-green	0.02249	< 0.05	yes	> 0.05/7	no
orange-green	0.05149	> 0.05	no	> 0.05/6	no
yellow-green	0.05906	> 0.05	no	> 0.05/5	no
blue-green	0.09751	> 0.05	no	> 0.05/4	no
red-yellow	0.1056	> 0.05	no	> 0.05/3	no
orange-yellow	0.2719	> 0.05	no	> 0.05/2	no
orange-red	0.5294	> 0.05	no	> 0.05/1	no

A3 *Astatotilapia burtoni* - males only (n = 11)

Friedman test

parameter	p-value
pecks	1.92*10-4
chases	0.025
pecks & chases	1.37*10-4

Wilcoxon signed-rank test

parameter	p-value	significant? (without Bonferroni correction)		significant? (with Bonferroni correction)	
**pecks**					
red-blue	0.005654	< 0.05	yes	> 0.05/10	no
red-green	0.008729	< 0.05	yes	> 0.05/9	no
yellow-blue	0.01368	< 0.05	yes	> 0.05/8	no
orange-red	0.01412	< 0.05	yes	> 0.05/7	no
yellow-green	0.01673	< 0.05	yes	> 0.05/6	no
orange-green	0.1003	> 0.05	no	> 0.05/5	no
orange-yellow	0.1213	> 0.05	no	> 0.05/4	no
orange-blue	0.1814	> 0.05	no	> 0.05/3	no
red-yellow	0.235	> 0.05	no	> 0.05/2	no
blue-green	1	> 0.05		> 0.05/1	no
**chases**					
red-green	0.01415	< 0.05	yes	> 0.05/10	no
orange-red	0.0754	> 0.05	no	> 0.05/9	no
red-blue	0.1604	> 0.05	no	> 0.05/8	no
red-yellow	0.3428	> 0.05	no	> 0.05/7	no
yellow-green	0.3491	> 0.05	no	> 0.05/6	no
yellow-blue	0.4982	> 0.05	no	> 0.05/5	no
orange-yellow	0.5248	> 0.05	no	> 0.05/4	no
orange-green	0.8923	> 0.05	no	> 0.05/3	no
orange-blue	1	> 0.05	no	> 0.05/2	no
blue-green	1	> 0.05	no	> 0.05/1	no
**pecks & chases**					
red-green	0.005857	< 0.05	yes	> 0.05/10	no
orange-red	0.007963	< 0.05	yes	> 0.05/9	no
yellow-blue	0.008909	< 0.05	yes	> 0.05/8	no
red-blue	0.01616	< 0.05	yes	> 0.05/7	no
yellow-green	0.02055	< 0.05	yes	> 0.05/6	no
orange-yellow	0.06769	> 0.05	no	> 0.05/5	no
orange-green	0.235	> 0.05	no	> 0.05/4	no
orange-blue	0.4164	> 0.05	no	> 0.05/3	no
red-yellow	0.4494	> 0.05	no	> 0.05/2	no
blue-green	0.8335	> 0.05	no	> 0.05/1	no

B1 *Pseudocrenilabrus multicolor* - males and females (n = 20)

Friedman test

parameter	p-value
pecks & chases	3.84*10-10

Wilcoxon signed-rank test

parameter	p-value	significant? (without Bonferroni correction)		significant? (with Bonferroni correction)	
**pecks**					
yellow-blue	0.0002061	< 0.05	yes	< 0.05/10	yes
orange-blue	0.0003161	< 0.05	yes	< 0.05/9	yes
red-blue	0.0004709	< 0.05	yes	< 0.05/8	yes
yellow-green	0.003481	< 0.05	yes	< 0.05/7	yes
orange-green	0.01214	< 0.05	yes	> 0.05/6	no
red-green	0.01139	< 0.05	yes	> 0.05/5	no
orange-yellow	0.05541	> 0.05	no	> 0.05/4	no
red-yellow	0.0839	> 0.05	no	> 0.05/3	no
blue-green	0.09751	> 0.05	no	> 0.05/2	no
orange-red	0.8139	> 0.05	no	> 0.05/1	no
**chases**					
red-blue	0.0003149	< 0.05	yes	< 0.05/10	yes
red-green	0.001603	< 0.05	yes	< 0.05/9	yes
orange-blue	0.001714	< 0.05	yes	< 0.05/8	yes
yellow-blue	0.004097	< 0.05	yes	< 0.05/7	yes
orange-red	0.01863	< 0.05	yes	> 0.05/6	no
red-yellow	0.04148	< 0.05	yes	> 0.05/5	no
orange-green	0.06971	> 0.05	no	> 0.05/4	no
blue-green	0.1268	> 0.05	no	> 0.05/3	no
yellow-green	0.1422	> 0.05	no	> 0.05/2	no
orange-yellow	0.8065	> 0.05	no	> 0.05/1	no
**pecks & chases**					
yellow-blue	0.0001421	< 0.05	yes	< 0.05/10	yes
red-blue	0.0001426	< 0.05	yes	< 0.05/9	yes
orange-blue	0.0002518	< 0.05	yes	< 0.05/8	yes
yellow-green	0.0002717	< 0.05	yes	< 0.05/7	yes
red-green	0.0005772	< 0.05	yes	< 0.05/6	yes
orange-green	0.01206	< 0.05	yes	> 0.05/5	no
orange-yellow	0.01842	< 0.05	yes	> 0.05/4	no
blue-green	0.04383	< 0.05	yes	> 0.05/3	no
orange-red	0.04509	< 0.05	yes	> 0.05/2	no
red-yellow	0.4812	> 0.05	no	> 0.05/1	no

B2 *Pseudocrenilabrus multicolor* - females only (n = 10)

Friedman test

parameter	p-value
pecks	3.76*10-5
chases	0.001
pecks & chases	2.20*10-6

Wilcoxon signed-rank test

parameter	p-value	significant? (without Bonferroni correction)		significant? (with Bonferroni correction)	
**pecks**					
red-blue	0.00897	< 0.05	yes	> 0.05/10	no
yellow-blue	0.00903	< 0.05	yes	> 0.05/9	no
orange-blue	0.00909	< 0.05	yes	> 0.05/8	no
red-green	0.01403	< 0.05	yes	> 0.05/7	no
yellow-green	0.01415	< 0.05	yes	> 0.05/6	no
orange-green	0.02812	< 0.05	yes	> 0.05/5	no
blue-green	0.3711	> 0.05	no	> 0.05/4	no
orange-red	0.675	> 0.05	no	> 0.05/3	no
orange-yellow	0.8923	> 0.05	no	> 0.05/2	no
red-yellow	1	> 0.05	no	> 0.05/1	no
**chases**					
red-blue	0.01403	< 0.05	yes	> 0.05/10	no
red-green	0.01427	< 0.05	yes	> 0.05/9	no
yellow-blue	0.02249	< 0.05	yes	> 0.05/8	no
orange-red	0.05802	> 0.05	no	> 0.05/7	no
yellow-green	0.05917	> 0.05	no	> 0.05/6	no
orange-blue	0.0925	> 0.05	no	> 0.05/5	no
red-yellow	0.1235	> 0.05	no	> 0.05/4	no
orange-green	0.3604	> 0.05	no	> 0.05/3	no
orange-yellow	0.4615	> 0.05	no	> 0.05/2	no
blue-green	0.4615	> 0.05	no	> 0.05/1	no
**pecks & chases**					
red-blue	0.001953	< 0.05	yes	< 0.05/10	yes
red-green	0.001953	< 0.05	yes	< 0.05/9	yes
yellow-blue	0.001953	< 0.05	yes	< 0.05/8	yes
yellow-green	0.001953	< 0.05	yes	< 0.05/7	yes
orange-blue	0.01427	< 0.05	yes	> 0.05/6	no
orange-red	0.02439	< 0.05	yes	> 0.05/5	no
red-yellow	0.04136	< 0.05	yes	> 0.05/4	no
orange-green	0.08528	> 0.05	no	> 0.05/3	no
blue-green	0.4185	> 0.05	no	> 0.05/2	no
orange-yellow	0.4469	> 0.05	no	> 0.05/1	no
yellow-green	0.5896	> 0.05	no	0.05/1	no

B3 *Pseudocrenilabrus multicolor* - males only (n = 10)

Friedman test

parameter	p-value
pecks	1.03*10-4
chases	0.006
pecks & chases	1.53*10-4

Wilcoxon signed-rank test

parameter	p-value	significant? (without Bonferroni correction)		significant? (with Bonferroni correction)	
**pecks**					
yellow-blue	0.008849	< 0.05	yes	> 0.05/10	no
orange-blue	0.01403	< 0.05	yes	> 0.05/9	no
orange-yellow	0.01782	< 0.05	yes	> 0.05/8	no
red-blue	0.02225	< 0.05	yes	> 0.05/7	no
red-yellow	0.02249	< 0.05	yes	> 0.05/6	no
yellow-green	0.08233	> 0.05	no	> 0.05/5	no
orange-green	0.1544	> 0.05	no	> 0.05/4	no
red-green	0.2719	> 0.05	no	> 0.05/3	no
blue-green	0.3711	> 0.05	no	> 0.05/2	no
orange-red	1	> 0.05	no	> 0.05/1	no
**chases**					
red-blue	0.008849	< 0.05	yes	> 0.05/10	no
orange-blue	0.01207	< 0.05	yes	> 0.05/9	no
red-green	0.05024	> 0.05	no	> 0.05/8	no
yellow-blue	0.1148	> 0.05	no	> 0.05/7	no
orange-red	0.1537	> 0.05	no	> 0.05/6	no
blue-green	0.1814	> 0.05	no	> 0.05/5	no
red-yellow	0.202	> 0.05	no	> 0.05/4	no
orange-green	0.3294	> 0.05	no	> 0.05/3	no
orange-yellow	0.4756	> 0.05	no	> 0.05/2	no
yellow-green	0.5896	> 0.05	no	> 0.05/1	no
**pecks & chases**					
orange-blue	0.007842	< 0.05	yes	> 0.05/10	no
yellow-blue	0.00903	< 0.05	yes	> 0.05/9	no
red-blue	0.009152	< 0.05	yes	> 0.05/8	no
orange-yellow	0.01427	< 0.05	yes	> 0.05/7	no
yellow-green	0.01437	< 0.05	yes	> 0.05/6	no
red-green	0.05041	> 0.05	no	> 0.05/5	no
orange-green	0.0921	> 0.05	no	> 0.05/4	no
blue-green	0.1003	> 0.05	no	> 0.05/3	no
red-yellow	0.2361	> 0.05	no	> 0.05/2	no
orange-red	0.7669	> 0.05	no	> 0.05/1	no

C1 *Julidochromis ornatus* - males and females (n = 20)

Friedman test

parameter	p-value
pecks & chases	1.34*10-6

Wilcoxon signed-rank test

parameter	p-value	significant? (without Bonferroni correction)		significant? (with Bonferroni correction)	
**pecks**					
yellow-green	0.02833	< 0.05	yes	> 0.05/10	no
yellow-blue	0.1039	> 0.05	no	> 0.05/9	no
red-yellow	0.1072	> 0.05	no	> 0.05/8	no
orange-yellow	0.2615	> 0.05	no	> 0.05/7	no
orange-green	0.3458	> 0.05	no	> 0.05/6	no
red-green	0.3711	> 0.05	no	> 0.05/5	no
orange-blue	0.5708	> 0.05	no	> 0.05/4	no
red-blue	0.7103	> 0.05	no	> 0.05/3	no
orange-red	0.8501	> 0.05	no	> 0.05/2	no
blue-green	1	> 0.05	no	> 0.05/1	no
**chases**					
yellow-blue	0.0006785	< 0.05	yes	< 0.05/10	yes
yellow-green	0.0006815	< 0.05	yes	< 0.05/9	yes
red-yellow	0.002849	< 0.05	yes	< 0.05/8	yes
orange-yellow	0.005581	< 0.05	yes	< 0.05/7	yes
orange-blue	0.007378	< 0.05	yes	< 0.05/6	yes
red-blue	0.01144	< 0.05	yes	> 0.05/5	no
orange-green	0.06387	> 0.05	no	> 0.05/4	no
blue-green	0.1165	> 0.05	no	> 0.05/3	no
red-green	0.2208	> 0.05	no	> 0.05/2	no
orange-red	0.7533	> 0.05	no	> 0.05/1	no
**pecks & chases**					
yellow-green	0.0004924	< 0.05	yes	< 0.05/10	yes
yellow-blue	0.0006513	< 0.05	yes	< 0.05/9	yes
red-yellow	0.003092	< 0.05	yes	< 0.05/8	yes
orange-yellow	0.01303	< 0.05	yes	> 0.05/7	no
orange-blue	0.01383	< 0.05	yes	> 0.05/6	no
red-blue	0.01852	< 0.05	yes	> 0.05/5	no
orange-green	0.0425	< 0.05	yes	> 0.05/4	no
red-green	0.04788	< 0.05	yes	> 0.05/3	no
blue-green	0.1163	> 0.05	no	> 0.05/2	no
orange-red	0.7798	> 0.05	no	> 0.05/1	no

C2 *Julidochromis ornatus* - females only (n = 11)

Friedman test

parameter	p-value
pecks	0.006
chases	8.53*10-5
pecks & chases	9.39*10-5

Wilcoxon signed-rank test

parameter	p-value	significant? (without Bonferroni correction)		significant? (with Bonferroni correction)	
**pecks**					
orange-green	0.02627	< 0.05	yes	> 0.05/10	no
yellow-blue	0.02627	< 0.05	yes	> 0.05/9	no
orange-red	0.1039	> 0.05	no	> 0.05/8	no
red-green	0.1039	> 0.05	no	> 0.05/7	no
orange-yellow	1	> 0.05	no	> 0.05/6	no
orange-blue	1	> 0.05	no	> 0.05/5	no
red-yellow	1	> 0.05	no	> 0.05/4	no
red-blue	1	> 0.05	no	> 0.05/3	no
yellow-green	1	> 0.05	no	> 0.05/2	no
blue-green	1	> 0.05	no	> 0.05/1	no
**chases**					
orange-yellow	0.00293	< 0.05	yes	< 0.05/10	yes
red-yellow	0.005889	< 0.05	yes	> 0.05/9	no
yellow-green	0.005922	< 0.05	yes	> 0.05/8	no
yellow-blue	0.006665	< 0.05	yes	> 0.05/7	no
orange-blue	0.02493	< 0.05	yes	> 0.05/6	no
red-blue	0.08293	> 0.05	no	> 0.05/5	no
blue-green	0.1232	> 0.05	no	> 0.05/4	no
orange-green	0.2084	> 0.05	no	> 0.05/3	no
orange-blue	0.02493	> 0.05	no	> 0.05/2	no
orange-red	1	> 0.05	no	> 0.05/1	no
**pecks & chases**					
red-yellow	0.003822	< 0.05	yes	< 0.05/10	yes
yellow-green	0.005857	< 0.05	yes	> 0.05/9	no
yellow-blue	0.007963	< 0.05	yes	> 0.05/8	no
orange-yellow	0.00903	< 0.05	yes	> 0.05/7	no
orange-blue	0.08273	> 0.05	no	> 0.05/6	no
blue-green	0.1824	> 0.05	no	> 0.05/5	no
red-blue	0.1846	> 0.05	no	> 0.05/4	no
orange-green	0.2072	> 0.05	no	> 0.05/3	no
red-green	0.3428	> 0.05	no	> 0.05/2	no
orange-red	1	> 0.05	no	> 0.05/1	no

C3 *Julidochromis ornatus* - males only (n = 9)

Friedman test

parameter	p-value
pecks	n.s.
chases	0.032
pecks & chases	0.022

Wilcoxon signed-rank test

parameter	p-value	significant? (without Bonferroni correction)		significant? (with Bonferroni correction)	
**pecks**					
orange-yellow	0.3711	> 0.05	no	> 0.05/10	no
orange-blue	0.3711	> 0.05	no	> 0.05/9	no
red-yellow	0.3711	> 0.05	no	> 0.05/8	no
red-blue	0.3711	> 0.05	no	> 0.05/7	no
orange-red	1	> 0.05	no	> 0.05/6	no
orange-green	1	> 0.05	no	> 0.05/5	no
red-green	1	> 0.05	no	> 0.05/4	no
yellow-green	1	> 0.05	no	> 0.05/3	no
blue-green	1	> 0.05	no	> 0.05/2	no
yellow-blue	NA	> 0.05	no	> 0.05/1	no
**chases**					
yellow-blue	0.05191	> 0.05	no	> 0.05/10	no
red-blue	0.05906	> 0.05	no	> 0.05/9	no
orange-blue	0.07969	> 0.05	no	> 0.05/8	no
yellow-green	0.08006	> 0.05	no	> 0.05/7	no
red-yellow	0.1508	> 0.05	no	> 0.05/6	no
orange-green	0.2359	> 0.05	no	> 0.05/5	no
red-green	0.2945	> 0.05	no	> 0.05/4	no
orange-yellow	0.3594	> 0.05	no	> 0.05/3	no
blue-green	0.5896	> 0.05	no	> 0.05/2	no
orange-red	0.8336	> 0.05	no	> 0.05/1	no
**pecks & chases**					
red-blue	0.03603	< 0.05	yes	> 0.05/10	no
yellow-blue	0.05191	> 0.05	no	> 0.05/9	no
red-green	0.05906	> 0.05	no	> 0.05/8	no
orange-blue	0.07969	> 0.05	no	> 0.05/7	no
yellow-green	0.08006	> 0.05	no	> 0.05/6	no
orange-green	0.1275	> 0.05	no	> 0.05/5	no
red-yellow	0.234	> 0.05	no	> 0.05/4	no
blue-green	0.4004	> 0.05	no	> 0.05/3	no
orange-yellow	0.4258	> 0.05	no	> 0.05/2	no
orange-red	0.8336	> 0.05	no	> 0.05/1	no
